# A Case Report and Literature Review of a Novel Mutation in the MAGED2 Gene of a Patient With Severe Transient Polyhydramnios

**DOI:** 10.3389/fped.2021.778814

**Published:** 2021-12-01

**Authors:** Xiaoxia Wu, Le Huang, Caiqun Luo, Yang Liu, Jianmin Niu

**Affiliations:** ^1^Cheeloo College of Medicine, Shandong University, Jinan, China; ^2^Affiliated Shenzhen Maternity and Child Healthcare Hospital, Southern Medical University, Shenzhen, China

**Keywords:** MAGED2, polyhydramnios, antenatal Bartter's syndrome, case report, mutation

## Abstract

**Background:** Polyhydramnios occurs frequently during pregnancy. Mutations in the MAGED2 gene can cause X-linked acute early-onset polyhydramnios with a severe but transient form of antenatal Bartter's syndrome.

**Case Presentation:** Here, we report a new novel frameshift mutation c.733_734delCT (p. Leu245GlufsTer4) in the MAGED2 gene (NM_177433.1) that caused prenatal polyhydramnios, but did not cause polyuria after birth. Follow-up was conducted for 2 months, and the baby's growth and development were normal, without polyuria and renal impairment. In addition, we identified all individuals with MAGED2 mutations reported in the literature before March 2021.

**Conclusion:** We report a new case with a novel variant of the MAGED2 gene that caused severe hydramnios but with a good result and summary clinical characteristics in a newborn with antenatal Bartter's syndrome caused by an MAGED2 mutation. Good prenatal diagnosis and genetic consultation can improve pregnancy monitoring and newborn management.

## Introduction

Excessive amniotic fluid (referred to as polyhydramnios) is a recurrent clinical problem. Polyhydramnios can be caused by an imbalance between fluid production and removal and is associated with increased perinatal adverse outcomes in fetuses. Overall, polyhydramnios occurs in approximately 1–2% of pregnancies, but the causes of polyhydramnios remain unknown in 30–60% of cases (1). A prenatal genetic diagnosis can help us identify the cause, allowing for appropriate management before and after birth.

There are only a few genetic diseases associated with polyhydramnios, such as Bartter's syndrome. Batter's syndrome is an autosomal recessive disease characterized by severe polyuria and sodium renal loss that was first described by Bartter et al., in 1962 (2). Phenotypically, Bartter's syndrome falls into two subgroups: antenatal Bartter's syndrome (aBS) and classical Bartter's syndrome. The typical features of aBS are reflected by the early onset of polyhydramnios and postnatal polyuria, which has been shown to be caused by mutations in the MAGED2 gene (melanoma-associated antigen D2) (3); the protein is not a solute carrier but a regulatory protein with function not fully understood. Here, we report a new case with a novel MAGED2 variant causing severe hydramnios but with a good neonatal outcome.

## Case Description

**Figure 1:**
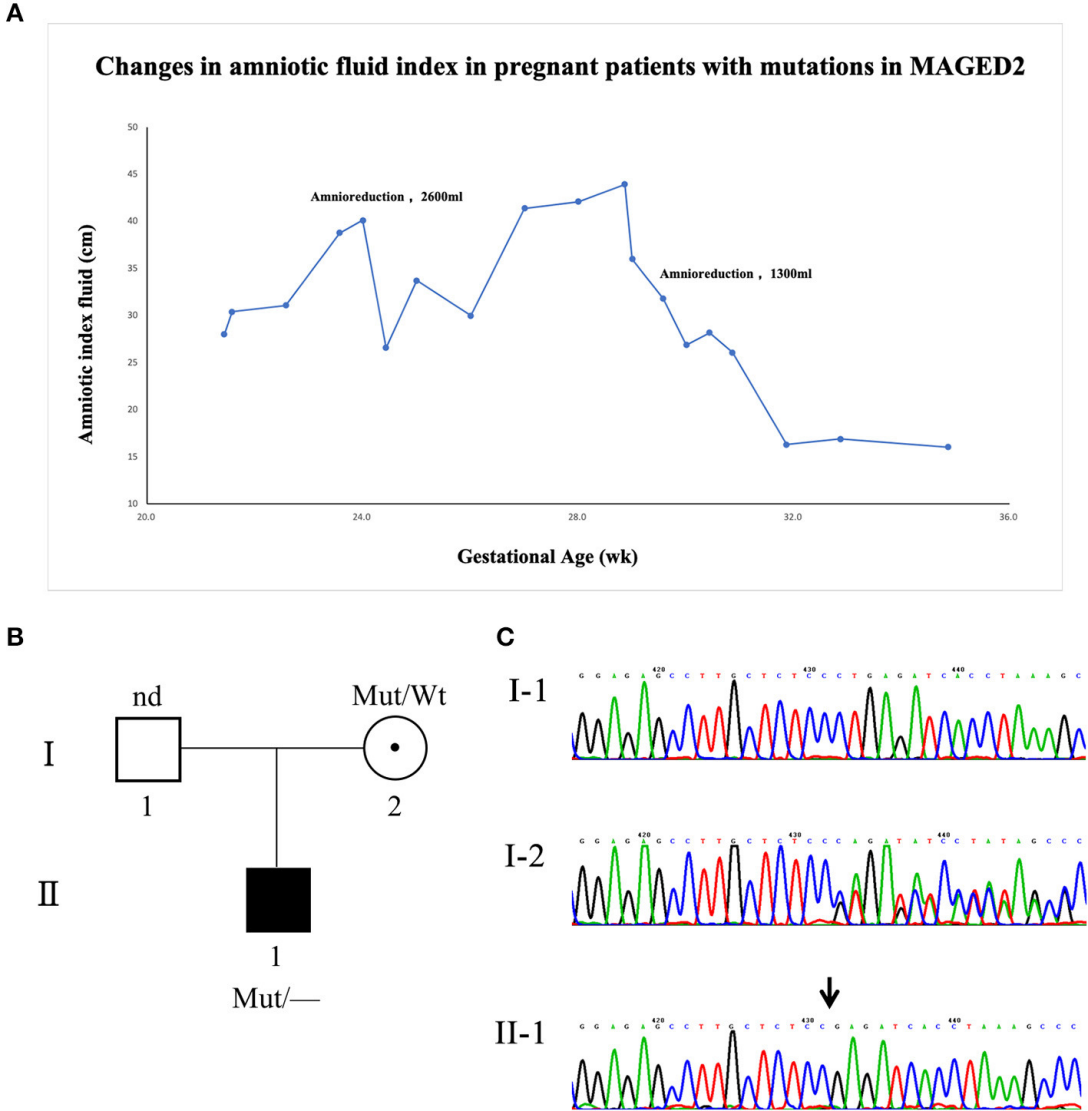
Clinical and genetic characteristics of the family with the MAGED2 mutation. **(A)** The changes in the amniotic fluid index during pregnancy for the patient with an MAGED2 mutation during pregnancy. **(B)** A pedigree diagram of the family. **(C)** Sanger analysis showed c.733_734delCT (p. Leu245GlufsTer4) of the MAGED2 (NM_177433.1) variant in the fetus; his mother carried the variant in the heterozygous state.

A primipara woman at the age of 27 years had regular checkups throughout pregnancy. The size of the fetus matched the gestational age, and there were no structural abnormalities in the fetus during pregnancy. Down's syndrome screening and Non-Invasive Prenatal Genetic Testing (NIPT) were negative. The 75-g oral glucose tolerance test (OGTT) showed that she did not have gestational diabetes. She was first diagnosed with polyhydramnios at 21 weeks of gestation. The amniotic index fluid before amnioreduction was 35.18 cm (range 28 to 43.94 cm). Two amnioreductions were required at 24 and 29 + 5 weeks of gestation, and the volume drained was 2,300 and 1,600 ml, respectively. Surprisingly, 1 week after the last amnioreduction, the amniotic index fluid returned to normal without any treatment. Prior to childbirth, she did not present with symptoms of polyhydramnios by ultrasound (Figure 1A), and the amniotic fluid was 850 ml at delivery. The genomic DNA of the fetus was extracted from amniotic fluid, and the parents' DNA was extracted from blood samples. Karyotype analysis and chromosomal microarray found no chromosomal abnormalities in the fetus. To further explore the pathogen, whole-exome sequencing (WES) was used identify a novel frameshift mutation c.733_734delCT (p. Leu245GlufsTer4) of the MAGED2 gene (NM_177433.1). Sanger analysis of the variant for the fetus showed that it came from his healthy mother, and his mother carried the variant in the heterozygous state (Figures 1B,C). She had one younger brother and one younger sister; both were in good condition at birth, and they did not have children at the time of this study.

**Figure 2:**
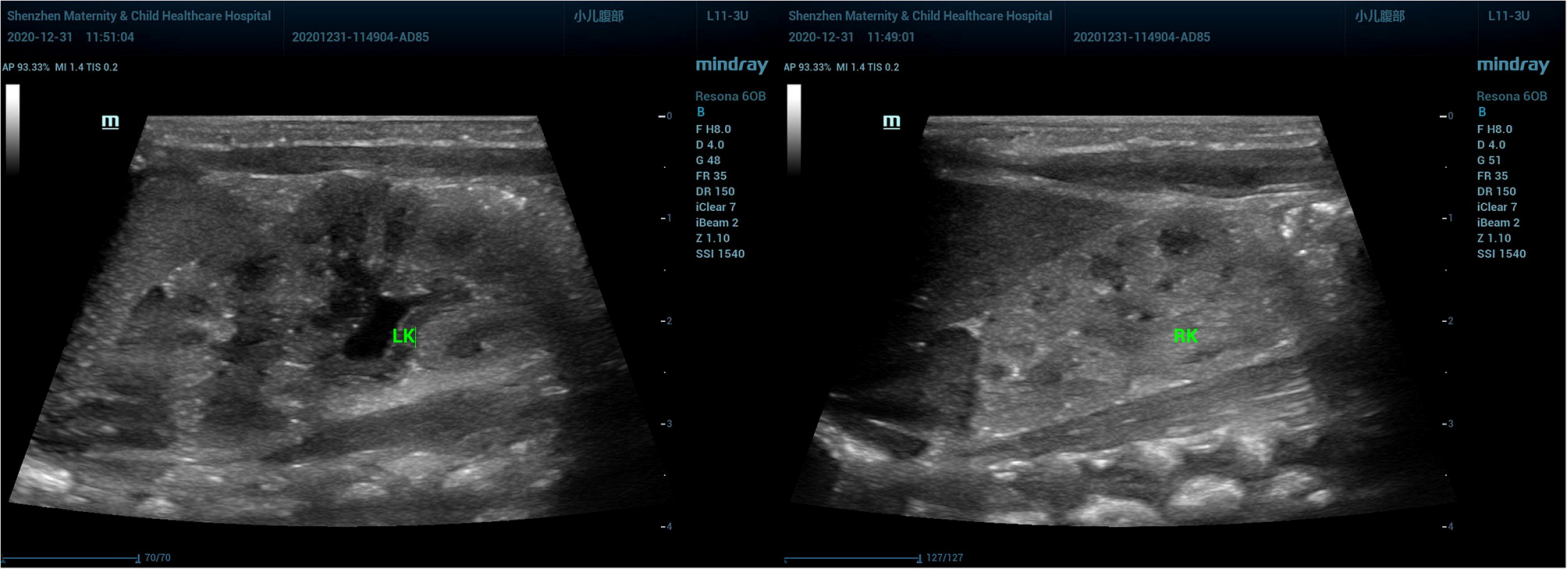
The kidney ultrasound of the male neonate. Normal kidneys and adrenal glands are shown.

A preterm healthy male neonate weighing 2,800 g was born *via* vaginal delivery at 35 + 2 weeks of gestation, with normal Apgar scores of 10 at 1 and 5 min; his birth length was 49 cm. Based on the Fenton Preterm Growth Chart (2013), the birth weight percentile was within the range between 50th and 90th percentile and the birth length percentile above the 90th percentile for the corresponding gestational age. Jaundice (TCB level 213/231 μmol/l, TBIL level 181.2 μmol/l, DBIL level 7 μmol/l) appeared 24 h after birth, and he was given phototherapy. His kidney ultrasound showed normal kidneys and adrenal glands; the left kidney was 4.61 × 2.37 cm, and the right kidney was 4.12 × 1.73 cm (Figure 2). Luckily, the baby did not develop polyuria, and he had a normal blood level of creatinine and a natremia. The boy was followed up for 7 days, 14 days, 1 month, and 2 months after birth and showed good growth and development, without polyuria and renal impairment (Table 1).

**Table 1:** Postnatal biochemical data of neonatal with MAGED2 mutation.

	**Normal value**	**Age**					
		**1 day**	**3 days**	**1 week**	**2 weeks**	**1 month**	**2 months**
**Urine data**							
Volume (ml/kg/h)		2.8	3.6	3.8	-	-	
Potassium-mmol/l	-	-	11.76	14.77	21.61	20.68	
Sodium-mmol/l	-	-	30.9	9.4	19.4	9.2	
Chloride-mmol/l	-	-	36.6	19.8	35.5	22.8	
Calcium-mmol/l	-	-	0.91	0.52	4.71	6.34	
Bicarbonates-mmol/l	-	-	4	5	<5	10.8	
Phosphorus-mmol/l	-	-	12.8	3.81	10.81	-	
Magnesium-mmol/l	-	-	1.15	0.6	4.12	1.29	
**Blood data**							
Potassium-mmol/l	3.5–5.3	3.97	4.08	4.58	5.26	4.58	4.64
Sodium-mmol/l	137–147	138.7	133.2	135.8	134.3	133.2	135.6
Chloride-mmol/l	96–108	106.4	104.9	106	104.3	102.5	105.6
Calcium-mmol/l	2.35–2.70	2.19	2.28	2.39	2.49	2.41	2.39
Bicarbonates-mmol/l	23–29	19.9	20.6	21.1	20.8	24.6	22.7
Phosphorus-mmol/l	1.45–2.10	2.36	1.93	2.11	2.10	1.81	2.09
Magnesium-mmol/l	0.5–0.9	0.91	0.9	0.72	0.81	0.73	0.85
Urea-mmol/l	2.1–7.1	3.45	1.98	2.22	2.3	0.8	1.8
Creatinine-μmol/l	54-110	62	54	44	37	25	21
Uric acid-μmol/l	286–518	273	161	142	233	113	121

## Literature Review

We attempted to identify all individuals with MAGED2 mutations reported in the literature before March 2021. We searched PubMed and Google Scholar using the following terms: “MAGED2 AND mutation AND (Polyhydramnios or Bartter's Syndrome)” to search all the literature to summarize all cases of polyhydramnios or Bartter's syndrome caused by an MAGED2 mutation. Since Laghmani et al. first reported that MAGED2 gene mutations can cause prenatal Bartter syndrome in 2016 (3), there have been 28 reports of MAGED2 gene mutations, including missense mutations, nonsense mutations, splice site mutations, and deletions (3–8) (Table 2).

**Table 2:** Genotype and clinical features of each MAGED2 mutations.

**Author**	**Year**	**Nucleotide** **(cDNA)**	**Protein**	**Type of** **mutation**	**Clinical** **characteristics**						
					**Polyhydramnios,** **onset (wk)**	**Gestational age at delivery (weeks)**	**Outcome**	**Polyuria**	**Polyuria, duration** **(weeks)**	**Nephrocalcinosis**	**Indometacin (years)**
Reinalter et al. (4)	1998	c.384_385TG	p. Val129fs	Frameshift	20	28	Survival	Yes	8	No	No
Laghmani et al. (3)	2016	c.1038C>G	p.Y346*	Nonsense	19	27;31	1 die,2 survival	Yes	5;1	Yes	1; No
		c.1462_73del12	p.488_491delEAAA	?	20	28	1 die,1 survival	Yes	5	No	No
		c.991-2A>G	New acceptor splice site	Splice site	19	34;33;31	3 survival	No; na; na	-	No	No; 9; 3
		c.1484C>G	p.A495D fs*39	Splice site	20	24	1 survival	Yes	6	Yes	1
		c.274dupA	p. T92Nfs*7	?	20	26	1 survival	Yes	4	Yes	No
		c.397A>T	p. Lys133Ter	Nonsense	19	27	1 survival	Yes	4	Yes	1.2
		c.386_87delTG	p.V129G fs*2	Splice site	na	30;29	1 die,1 survival	Yes	1	Yes	2.8
		c.1336 C>T	p. R446C	Missense	19	22	1 die	No	-	-	-
		c.847- 9C>G	p. A283Sfs*8	Splice site	19	29	1 survival	No	-	-	-
Legrand et al. (5)	2017	c.607C.T	p. Arg203Ter	Nonsense		-	Survival	Yes			
		c.842_843dup	p. Arg282GlyfsTer7	Frameshift	yes	-	Die after 1 years old thrombocytopenia	Yes			
	-	c.967C.T	p. Arg323Ter	Nonsense		-	Survival	Yes			
		c.967dup	p. Arg323ProfsTer18	Frameshift		-	Survival	Yes			
		c.10851 + G>A	p.?	Splice (frameshift)		-	Survival	Yes			
		c.12711 + G>A	p.?	Splice (in frame)		-	Die after 1 years old, leukomalacia severe dehydration, and hyper natremia	Yes			
		c.1336C>T	p. Arg446Cys	Missense	no	33	Survival	Yes			
		c.1337G>A	p. Arg446His	Missense		-	Survival	Yes			
		c.1366G>T	p. Val456Phe	Missense	no	-	Survival	Yes			
		c.1384_13864 + del	p.?	Splice (frameshift)	yes	-	Survival	Yes			
		c.1420C>T	p. Gln474Ter	Nonsense		-	Survival	Yes			
		c.1458_1466del	p. Glu488_Ala490del	In frame deletion		-	Survival	Yes			
		c.1464_1475del	p. Ala490_Ala493del	In frame deletion		-	Survival	Yes			
		c.1515_1516dup	p. Gly506ValfsTer71	Frameshift		-	Survival	Yes			
		Complete deletion	p.?	Large deletion		-	Survival	Yes			
Meyer et al. (6)	2018	-	-	-	21	29	Survival	Yes			No
Arthuis et al. (8)	2018	c.823delG	p. Asp275Metfs 13	Frameshift	19	36	Survival	No		No	No
Yang et al. (9)	2019	Complete deletion	-	Large deletion	20	na	3 die, 1 na				
Our case		c.733_734delCT	p. Leu245GlufsTer4	Frameshift	21	35+2	Survival	No		No	No

## Discussion and Conclusions

Compared with other gene mutation types, mutations in the MAGED2 gene cause X-linked acute early-onset polyhydramnios with a severe but transient form of aBS (9). Most cases occurred in males who experienced severe symptoms of polyhydramnios prenatally, and surviving infants showed symptoms of polyuria after birth, but the symptoms disappeared spontaneously during months 1–18. Currently, long-term follow-up results are good. The MAGED2 gene (located on Xp11.21) encodes MAGE-D2, which is shown in the thick ascending limb of the loop of Henle and the distal tubules, and plays an important role in fetal and adult kidneys. These severe clinical characteristics can also be observed in females because of extremely skewed X-inactivation (5). The spontaneous remission of symptoms with MAGE-D2 related to Bartter syndrome might be related to the following two reasons: (1) the reduction in Gs-α activity might be equilibrated by the sensitivity to vasopressin, and (2) hypoxia in renal fetal tissue would lead to greater degradation of NCC and NKCC2, which could be improved after delivery (3, 10). Therefore, the mutation of this gene can cause an imbalance in amniotic fluid during pregnancy and affects the reabsorption of fetal kidney salt.

Our case is consistent with the earlier clinical features of polyhydramnios caused by an MAGED2 gene mutation. The fetus was born as a male baby, and this case is also consistent with the x-linked recessive inheritance of the MAGED2 gene. Our report is a new mutation; after two amnioreductions in the second trimester, the woman's polyhydramnios symptoms were relieved spontaneously until the time of delivery, and the symptoms after birth were mild. It is speculated that the mutation at the altered site has a good pregnancy outcome, which may be related to genetic polymorphism or spatiotemporal specificity. Further explorations and fundamental studies are necessary to validate this hypothesis and to understand the transient character of this mutation.

In summary, we have reported the prenatal Bartter's syndrome caused by a new mutation in the MAGED2 gene, and the follow-up neonatal outcome was good. Bartter's syndrome caused by this gene mutation may lead to avoiding unnecessary measures during pregnancy and for premature babies and provide information to couples with regarding neonatal outcomes, which is the key to improving prenatal counseling services. It also provides evidence for future pregnancies. The identification of the MAGED2 gene mutation of antenatal Bartter's syndrome is important due to the X-linked nature of the disease and for management of fetus/neonate before and after delivery. It is also important to be informed of the disease to avoid treating while the patient has recovered.

## Data Availability Statement

The original contributions presented in the study are included in the article/supplementary materials, further inquiries can be directed to the corresponding author.

## Ethics Statement

Written informed consent was obtained from the minor(s)' legal guardian/next of kin for the publication of any potentially identifiable images or data included in this article.

## Author Contributions

XXW and LH drafted the manuscript. CQL and YL drew the diagrams and collected the patient data. JMN revised the manuscript. All authors read and approved the final manuscript.

## Conflict of Interest

The authors declare that the research was conducted in the absence of any commercial or financial relationships that could be construed as a potential conflict of interest.

## Publisher's Note

All claims expressed in this article are solely those of the authors and do not necessarily represent those of their affiliated organizations, or those of the publisher, the editors and the reviewers. Any product that may be evaluated in this article, or claim that may be made by its manufacturer, is not guaranteed or endorsed by the publisher.

## References

[1] Magann EF Chauhan SP Doherty DA Lutgendorf MA Magann MI Morrison JC. review of idiopathic hydramnios and pregnancy outcomes. Obstet Gynecol Surv. (2007) 62:795–802. 10.1097/01.ogx.0000290349.58707.e0 18005456

[2] Bartter FC Pronove P Gill JR Jr Maccardle RC. Hyperplasia of the juxtaglomerular complex with hyperaldosteronism and hypokalemic alkalosis A new syndrome. Am J Med. (1962) 33:811–28. 10.1016/0002-9343(62)90214-0 13969763

[3] Laghmani K Beck BB Yang SS Seaayfan E Wenzel A Reusch B. Polyhydramnios, transient antenatal bartter's syndrome, and MAGED2 mutations. N Engl J Med. (2016) 374:1853–63. 10.1056/NEJMoa1507629 27120771

[4] Reinalter S Devlieger H Proesmans W. Neonatal Bartter syndrome: spontaneous resolution of all signs and symptoms. Pediatr Nephrol. (1998) 12:186–8. 10.1007/s004670050433 9630034

[5] Legrand A Treard C Roncelin I Dreux S Bertholet-Thomas A Broux F. Prevalence of novel MAGED2 mutations in antenatal bartter syndrome. Clin J Am Soc Nephrol. (2018) 13:242–50. 10.2215/CJN.05670517 29146702 PMC5967426

[6] Meyer M Berrios M Lo C. Transient antenatal bartter's syndrome: a case report. Front Pediatr. (2018) 6:51. 10.3389/fped.2018.00051 29594084 PMC5857533

[7] Yang K Huo X Zhang Y Zhang M Gao Y Wu D. Genetic analysis of a pedigree affected with Bartter's syndrome. Zhonghua Yi Xue Yi Chuan Xue Za Zhi. (2019) 36:701–3. 31302915 10.3760/cma.j.issn.1003-9406.2019.07.011

[8] Arthuis CJ Nizon M Komhoff M Beck BB Riehmer V https://pubmed.ncbi.nlm.nih.gov/?term=Bihou%C3%A9e+T&cauthor_id=29893154 Bihouée T A step towards precision medicine in management of severe transient polyhydramnios: MAGED2 variant. J Obstet Gynaecol. (2019) 39:395–7. 10.1080/01443615.2018.1454415 29893154

[9] Komhoff M Laghmani K. MAGED2: a novel form of antenatal Bartter's syndrome. Curr Opin Nephrol Hypertens. (2018) 27:323–8. 10.1097/MNH.0000000000000422 29677005

[10] Quigley R Saland JM. Transient antenatal Bartter's Syndrome and X-linked polyhydramnios: insights from the genetics of a rare condition. Kidney Int. (2016) 90:721–3. 10.1016/j.kint.2016.07.031 27633862

